# Compend of Anatomy

**Published:** 1881-01

**Authors:** 


COMPEND OF ANATOMY. By JOHN B. ROBERTS, A. M., M. D., Lecturer on Anatomy, etc., Philadelphia School of Anatomy, etc. Philadelphia: C. C. Roberts & Co. 1880. 16mo, pp. 191.

This excellent little book is an abbreviation of our standard works on anatomy. It is designed for use in the dissecting room—for those who are preparing for examinations, and for hasty reference. It is well arranged; much of it is in tabular form, especially the portion devoted to the muscles and nerves. Many Latin names have been substituted by appropriate English names. In short, it is an anatomical brief admirably adapted to the purposes for which it is designed.

